# Supplementary material for: Enabling Ab Initio Molecular Dynamics under Bias: The CP2K+SMEAGOL Interface for Integrating Density Functional Theory and Non-Equilibrium Green Functions
Source: J Chem Theory Comput. 2024 Jul 16;20(15):6772–80. doi: 10.1021/acs.jctc.4c00371 (PMC11325543; doi:10.1021/acs.jctc.4c00371)
Supplement: Supplementary file 1 — ct4c00371_si_001.pdf [file ct4c00371_si_001.pdf]

Supplementary Information: Enabling Ab-Initio Molecular Dynamics under Bias: The CP2K+SMEAGOL Interface for Integrating Density Functional Theory and Non-Equilibrium Green Functions

Christian S. Ahart,[†] Sergey K. Chulkov,[‡] and Clotilde S. Cucinotta[*,†]

†*Imperial College London, Department of Chemistry and Thomas Young Centre, Molecular Sciences Research Hub, London W12 0BZ, UK*

‡*University of Lincoln, School of Mathematics and Physics, Lincoln LN67TS, UK*

E-mail: c.cucinotta@imperial.ac.uk

1 Supplementary Information

1.1 Zero bias forces

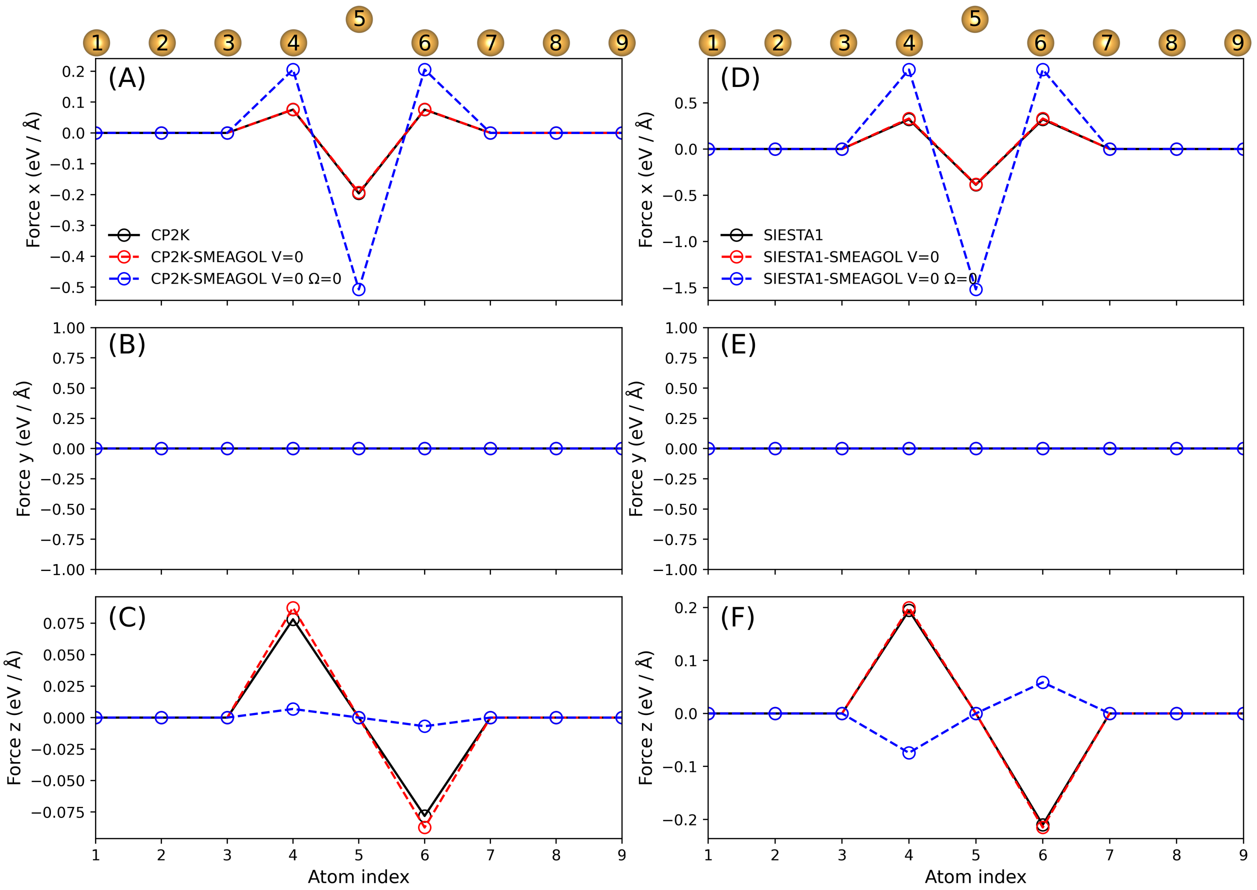

Figure 1: Zero bias tests for an infinite Au wire. (A)-(C) Atomic forces calculated with CP2K+SMEAGOL. (D)-(F) Atomic forces calculated with SIESTA+SMEAGOL.

1.2 Parallel-plate capacitor

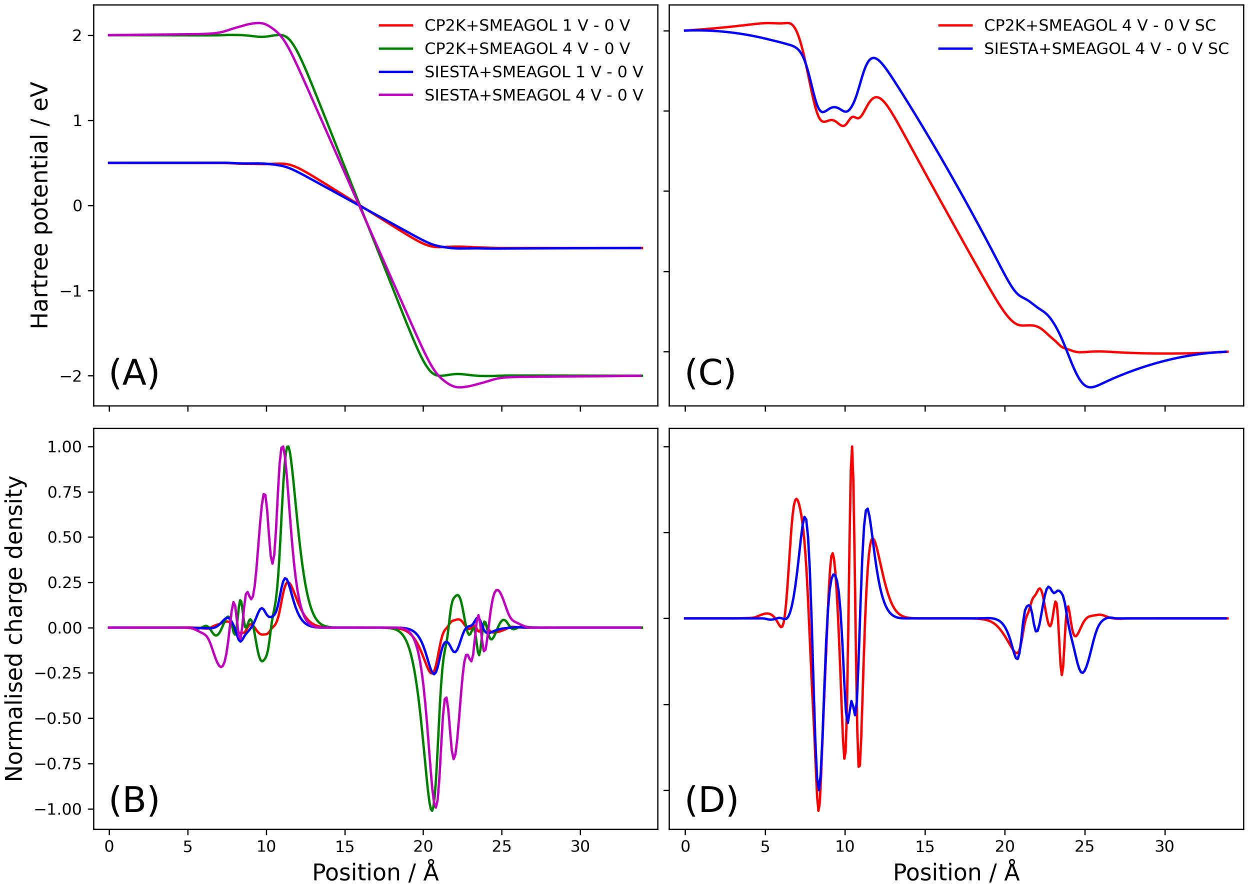

Figure 2: Finite bias tests for a parallel-plate capacitor. (A) Planar average of the Hartree potential difference calculated with and without an applied bias using CP2K+SMEAGOL and SIESTA+SMEAGOL. (B) Planar average of the charge density difference calculated with and without an applied bias. (C) Planar average of the Hartree potential difference calculated with and without an applied bias using a single contour evaluation of the Green's function, leading to an incorrect occupation of bound states within the bias window. (D) Planar average of the charge density difference calculated with and without an applied bias using a single contour evaluation of the Green's function.

1.3 Structure of solvated Au wire system

There are a number of requirements for a DFT-NEGF calculation that lead to the large system size of the solvated Au wire. The key requirement is that the semi-infinite leads must be periodic and reach bulk conditions, such that for the Au(111) surface the number of layers must be a multiple of three to reproduce the ABC periodicity. There are also constraints regarding the size of the leads both parallel and perpendicular to the transport direction. Parallel to the transport direction it is required that only nearest unit cells interact. Perpendicular to the transport direction this is not a requirement, however if multiple cell replicas must be stored in memory this significantly increases the computational cost of performing the DFT-NEGF calculation. SIESTA uses short-range truncated basis sets, which typically do not exceed 4 Å and as such these system size requirements are usually satisfied with only three atoms in each direction. CP2K however does not use truncated basis sets, and a typical basis set can exceed 10 Å before decaying to zero. As such, large system sizes are generally required in CP2K+SMEAGOL.

For the Au(001) systems studied in Section 3.2 and Sections 3.3 we used four layers (ABAB), however for the solvated Au wire the number of layers must be a multiple of three. Three layers does not satisfy the requirement that only nearest unit cells interact, and as such we must use six layers (ABCABC). Perpendicular to the transport direction we also use six layers, as it was found that it is computationally more efficient to increase the size of the leads than to store additional cell images in memory.

The requirement of reaching bulk conditions in the semi-infinite leads requires the introduction of an additional screening region between the different basis sets of the extended molecule and the leads. As such we introduce another three layers (ABC) of single-ζ Au(6s) atoms.

1.4 Molecular dynamics energy conservation

The hydrogen dimer presents one of the simplest benchmarks for examining energy convergence, for which we use the previously studied Au-H_2-Au junction. The same system setup is used as in Section 3.3, but where all Au atoms are constrained so that only the 2 H atoms move during the molecular dynamics. The system is first equilibrated for 1 ps with CP2K DFT-MD NVT, where the H_2 adsorbs onto the the right-most Au atom. We then perform an additional 1 ps of NVE MD using both CP2K and CP2K+SMEAGOL with an applied bias between V=0 to V=10. Fig. 3 shows the average drift of the conserved energy as a function of the applied bias up to V=6 using both a single contour and weighted double contour evaluation of the Green's function. It is found that while there are large instantaneous oscillations in the total energy, there is no long-term energy drift for all applied bias where the system is stable. At an applied bias of V=6 only the dynamics with the weighted double contour evaluation of the Green's function are stable, and for an applied bias of V=8 and V=10 the dynamics are not stable for either a single or weighted double contour evaluation. As such, we find energy conserving molecular dynamics of the hydrogen dimer for all experimentally relevant values of the applied bias.[1]

We also consider energy conservation for a larger system, the solvated Au wire with 1338 total atoms in Section 3.4. Fig. 4 shows the average drift of the conserved energy as well as the temperature for both CP2K and CP2K+SMEAGOL NVE MD. For CP2K there is no observable energy drift, while for CP2K-SMEAGOL with V=0 there is an energy drift of around 2×10^{-5} H/atom/ps. There is no change in energy drift when a small bias of V=0.1 is applied, shown by the overlap of the grey blue lines in Fig. 4. When a large bias of V=1 is applied across the system the energy drift increases to around 1×10^{-4} H/atom/ps. We note that given the small length of the CP2K+SMEAGOL MD performed of 100 fs, it is possible that the is not a long-term energy drift but instead a short-term energy oscillation as observed for the Au-H_2-Au junction. Regardless, while the energy drift is large it is comparable to other non-equilibrium or non-adiabatic MD such as CDFT-MD.[2]

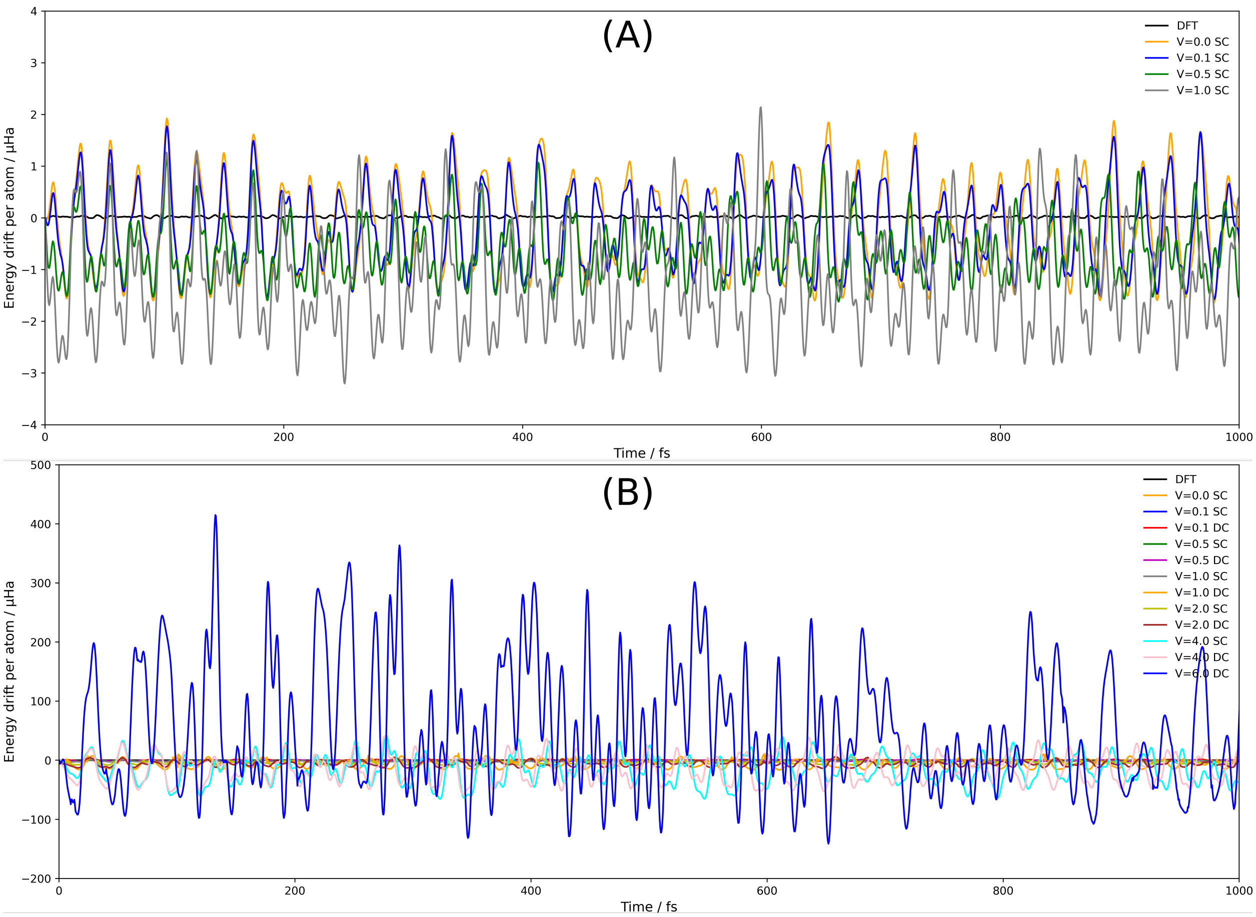

Figure 3: (A) Drift of the conserved energy in CP2K and CP2K+SMEAGOL NVE MD for an applied bias between V=0 and V=1, (B) between V=0 and V=6 for the Au-H_2-Au junction. SC refers to single contour evaluation of the Green's function and DC a weighted double contour evaluation, important for large applied bias.

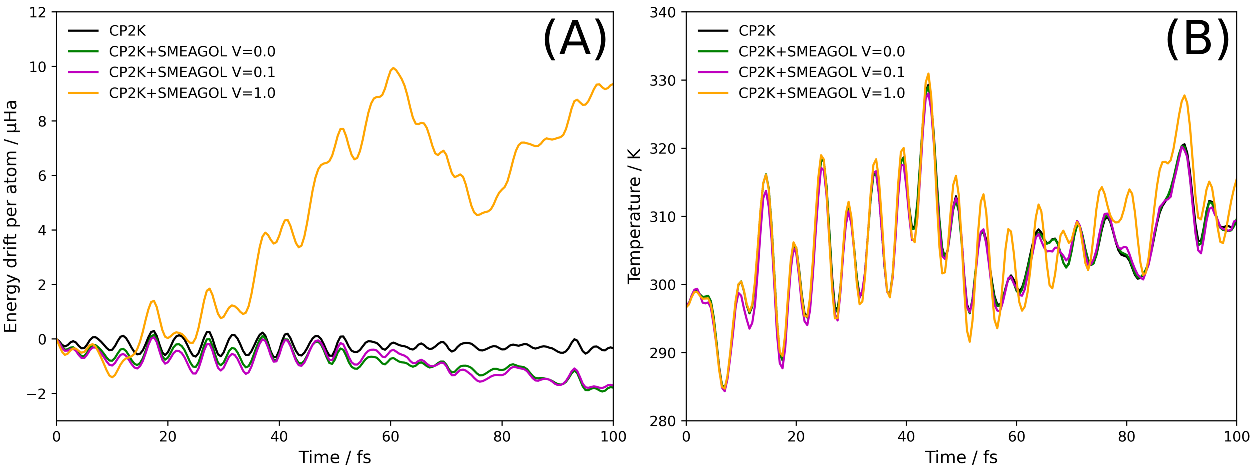

Figure 4: (A) Drift of the conserved energy during CP2K and CP2K+SMEAGOL MD for the solvated Au wire. (B) Temperature during CP2K and CP2K+SMEAGOL MD.

1.5 Density of states for solvated Au wire

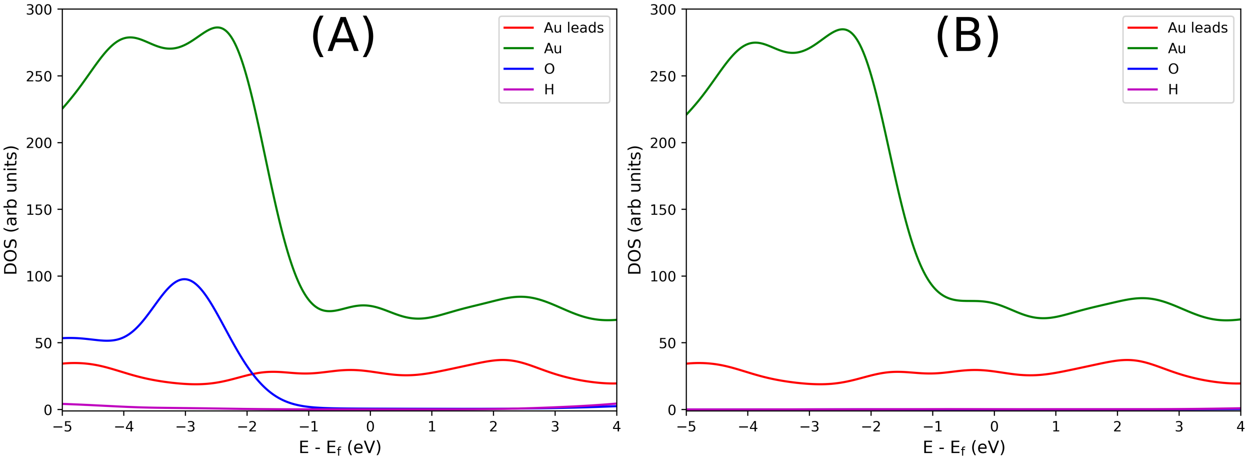

Figure 5: Projected density of states for the solvated Au wire (A) and with water molecules replaced with dummy atoms (B) calculated with CP2K at zero bias. The peak in the density of states for oxygen between -4 and -2 eV is consistent with the peak in the transmission between -4 and -2 eV found for the solvated Au wire (Figure 6).

1.6 Au-Melamine-Au junction

In this additional example we reproduce the change of activation barrier for switching between the C1 and C2 structures of melamine absorbed onto an Au(001) surface.[3,4] The C1 structure is shown in Figure 6, with an arrow indicating the 180 degree rotation of the N-H bond to form the C2 structure.

Table 1 shows the energies of the optimised GS, C1 and C2 structures in CP2K as well as reference calcualtions performed using SIESTA, in addition to literature calculations using SEISTA[3] and VASP[4] . The transition state structure TS2 was obtained by performing a CI-NEB[5] calculation starting from the optimised C1 and C2 geometries with the transition state guess generated manually. CP2K does not currently support frozen atoms with NEB, and therefore the atoms that should be frozen are allowed to move during the CI-NEB calculation, and then replaced with their unoptimized positions. As such, the TS2 structure energy is slightly higher than the reference calculations.

Figure 6 shows the change of activation barrier C1-TS2 and C2-TS2 under finite bias, with qualitative agreement between CP2K+SMEAGOL and SIESTA+SMEAGOL. The change in C1-TS2 from -0.5 to 0.5 V in CP2K is 8 meV and in SIESTA+SMEAGOL is 14 meV, reasonable agreement when considering that these energy differences are on the same order of magnitude as numerical error in a standard DFT calculation.

Table 1: Energy of GS, C1, TS2 and C2 relative to the energy of GS.

	Au CP2K / eV	Au SIESTA / eV	Cu SIESTA[3]/ eV	Cu VASP / eV[4]
GS	0.00	0.00	0.00	0.00
C1	0.77	0.71	0.72	0.71
TS2	2.01	1.82	1.70	1.77
C2	1.05	0.95	0.95	0.95

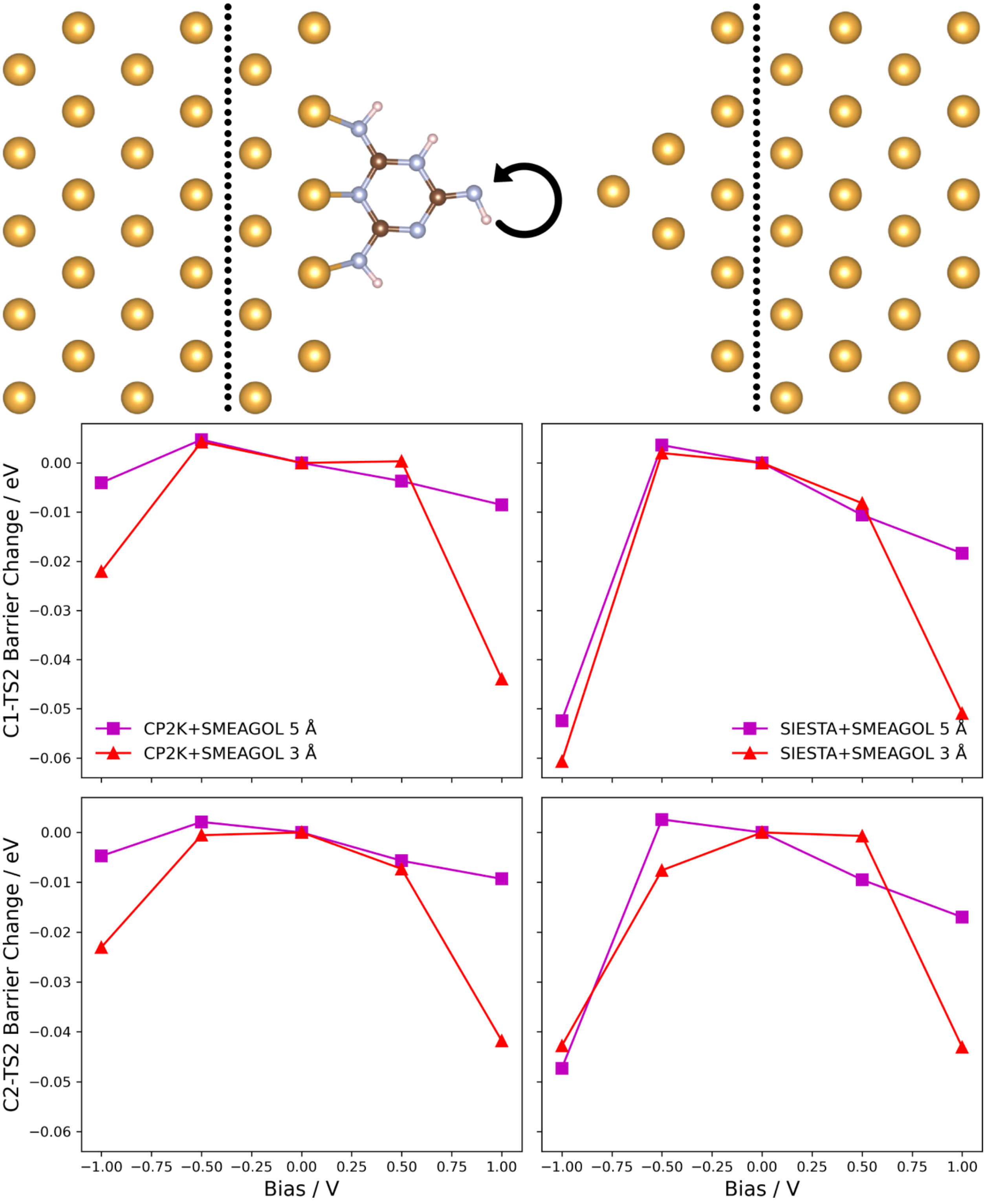

Figure 6: Change of activation barrier C1-TS2 and C2-TS2 under finite bias. Calculations are performed for CP2K+SMEAGOL (left) and SIESTA1+SMEAGOL (right).

References

(1) Reed, M. A.; Zhou, C.; Muller, C. J.; Burgin, T. P.; Tour, J. M. Conductance of a Molecular Junction. Science **1997**, 278, 252–254.

(2) Ahart, C. S.; Rosso, K. M.; Blumberger, J. Implementation and Validation of Constrained Density Functional Theory Forces in the CP2K Package. Journal of Chemical Theory and Computation **2022**,

(3) Ohto, T.; Rungger, I.; Yamashita, K.; Nakamura, H.; Sanvito, S. Ab Initio Theory for Current-Induced Molecular Switching: Melamine on Cu(001). Phys. Rev. B **2013**, 87, 205439.

(4) Pan, S.; Fu, Q.; Huang, T.; Zhao, A.; Wang, B.; Luo, Y.; Yang, J.; Hou, J. Design and Control of Electron Transport Properties of Single Molecules. Proc. Natl. Acad. Sci. U.S.A. **2009**, 106, 15259–15263.

(5) Henkelman, G.; Uberuaga, B. P.; Jónsson, H. A Climbing Image Nudged Elastic Band Method for Finding Saddle Points and Minimum Energy Paths. The Journal of Chemical Physics **2000**, 113, 9901–9904.
